# A Sex- and Gender-Based Analysis of Adverse Drug Reactions: A Scoping Review of Pharmacovigilance Databases

**DOI:** 10.3390/ph15030298

**Published:** 2022-02-28

**Authors:** Andreea C. Brabete, Lorraine Greaves, Mira Maximos, Ella Huber, Alice Li, Mê-Linh Lê

**Affiliations:** 1 Centre of Excellence for Women’s Health, Vancouver, BC V6H 3N1, Canada; lgreaves@cw.bc.ca (L.G.); mmaximos@uwaterloo.ca (M.M.); ellahuber95@gmail.com (E.H.); aliceli79@gmail.com (A.L.); 2 School of Population and Public Health, Faculty of Medicine, University of British Columbia, Vancouver, BC V6T 1Z3, Canada; 3 Woodstock Hospital, Woodstock, ON N44 0A4, Canada; 4 School of Pharmacy, University of Waterloo, Kitchener, ON N2G 1C5, Canada; 5 Neil John Maclean Health Sciences Library, University of Manitoba, Winnipeg, MB R3M 3M1, Canada; me-linh.le@umanitoba.ca

**Keywords:** adverse drug reactions, sex, gender, SGBA+, lifecycle management of drugs, pharmacovigilance

## Abstract

Drug-related adverse events or adverse drug reactions (ADRs) are currently partially or substantially under-reported. ADR reporting systems need to expand their focus to include sex- and gender-related factors in order to understand, prevent, or reduce the occurrence of ADRs in all people, particularly women. This scoping review describes adverse drug reactions reported to international pharmacovigilance databases. It identifies the drug classes most commonly associated with ADRs and synthesizes the evidence on ADRs utilizing a sex- and gender-based analysis plus (SGBA+) to assess the differential outcomes reported in the individual studies. We developed a systematic search strategy and applied it to six electronic databases, ultimately including 35 papers. Overall, the evidence shows that women are involved in more ADR reports than men across different countries, although in some cases, men experience more serious ADRs. Most studies were conducted in higher-income countries; the terms adverse drug reactions and adverse drug events are used interchangeably, and there is a lack of standardization between systems. Additional research is needed to identify the relationships between sex- and gender-related factors in the occurrence and reporting of ADRs to adequately detect and prevent ADRs, as well as to tailor and prepare effective reporting for the lifecycle management of drugs.

## 1. Introduction

An adverse drug reaction (ADR) is a harmful effect suspected to be caused by a drug [1]. The detection, assessment, and reporting of ADRs are important pharmacovigilance activities conducted to understand and prevent the occurrence of ADRs. However, regulators in many countries rely on voluntary reports of ADRs by healthcare providers and the public to pharmacovigilance databases in order to detect concerns regarding drug safety in the post-marketing phase of the lifecycle management of drugs [2]. This process is vulnerable to under-reporting of ADRs, with fewer than 5% of ADRs being reported, even in jurisdictions where reporting is mandatory [3,4]. 

Reporting systems in different countries are not uniform in their information requirements. In Canada, for example, ADR reporting is voluntary with the exception of hospitals, which have been subject to mandatory reporting since 2019 [5]. In a systematic review on adverse drug event (ADE) reporting systems, there was great variability in data fields and corresponding response options [6]. However, among the 108 ADE reporting systems identified, only 10 included information on age and sex [6]. Without robust ADR reporting systems with comprehensive information on sex, gender, and intersectional (age, socioeconomic status, race, ethnicity, and/or geographic location) factors, ADRs that are more common among certain groups, such as women, may remain undetected for years, increasing the possibility of unanticipated risks. These risks may be categorized as idiosyncratic by clinicians but may have a basis in physiologic and pharmacologic facets. Despite the limitations of the spontaneous ADR reporting systems, they are still the most used method within pharmacovigilance centers across many countries, as they provide a basis of understanding of signals related to potential ADRs to be further investigated [7].

The patterns of drug withdrawals from the market have been starkly variable. For instance, the US Government Accountability Office found that among the 10 prescription drugs withdrawn from the market between 1997 and 2001, eight of them had greater health risks for women than for men. While four drugs were found to have more adverse events in women because they were prescribed more often to women than men, the other four had more adverse events in women although they were prescribed to both women and men [8]. 

With these inequities and limitations in mind, this review synthesizes and assesses the evidence on ADRs using sex- and gender-based analysis plus (SGBA+). SGBA+ considers and analyzes how sex- and gender-related factors are integrated into research, policy, or health programs in order to revisit or identify the influence of sex-related factors (anatomy, physiology, genetics, and other biological characteristics) and gender-related factors (roles, norms, identities, and institutional patterns) affecting humans [9]. Sex and gender are not independent of other characteristics such as age, race, or ethnicity, as they can interact with each other and with other characteristics to influence health outcomes, as indicated by the “plus” factor of SGBA+ [10].

## 2. Background

The uneven representation of women and the lack of focus on sex in ADR identification begin early in the drug development cycle. After thalidomide and diethylstilbestrol (DES) teratogenicity associations were made in the 1950s and 1960s with major consequences to the offspring of women exposed to these drugs during pregnancy, many governments decided to adopt policies excluding pregnant women from clinical trials. This measure was soon extended to all women of reproductive age as researchers started considering that all females between the time of first menstruation and menopause as “potentially pregnant” [11]. During the 1990s, women’s health advocates requested more inclusion in clinical trials, as it was recognized that drug outcomes need to be assessed in both sexes [12]. 

However, even when women are included in clinical trials, the results are often not analyzed and reported for males/men and females/women separately. For example, in a study that analyzed 100 Canadian-led or -funded randomized controlled trials (RCTs), Welch et al. (2017) found that 98% of studies included sex in the description of sociodemographic characteristics of the participants, while only 6% conducted a subgroup analysis across sex, and only 4% reported sex-disaggregated data [13]. Failing to include a sex- and gender-based analysis of drug outcomes has important and serious clinical consequences for individuals or subgroups of patients, and in particular for women [14].

One way to predict and account for sex-specific differences in ADRs is to include data on pharmacokinetic (PK) and pharmacodynamic (PD) processes that affect or determine the impact of prescribed drugs. Sex-related factors affect PK, including the absorption, distribution, metabolism, and elimination of drugs. Sex differences regarding drug clearance are linked to sex-related factors in the expression of metabolic enzymes [15,16], and renal clearance of drugs is decreased in females because of a lower glomerular filtration rate compared to males [17]. Additionally, females have lower gastric emptying times, gastric pH, lean body mass, and hepatic clearance but higher plasma volume, BMI, and body fat, which, when coupled with a difference in Cytochrome P450 enzyme activity, can all contribute to a difference in the rate of drug metabolism compared with males [18]. There is evidence that sex differences in PK positively predict sex differences in ADRs [19]. In essence, if a female-biased PK sex difference is identified, there is a high probability that a clinically identifiable female-biased ADR will co-occur [19].

Gender-related factors also affect the reporting of ADRs by and about men and women. The reporting of ADRs includes assigning signs or symptoms to a drug, and these are perceived in different ways by women and men [20]. Compared to men, women show greater interest in and report more active seeking of health-related information, resulting in the receipt of more informal health-related information from close family members, other kin, and friends/workmates [21].

Hence, the purpose of this scoping review was to synthesize sex- and gender-related ADR outcomes and analyze how sex and gender are considered in analyses and reporting using data from national pharmacovigilance systems from countries such as the United States of America (USA), Australia, and those in the European Union (EU) (including the United Kingdom (UK)).

## 3. Methods

We followed Arksey and O’Malley’s [22] framework and the refinements suggested by Levac et al. [23] and Tricco et al. [24] for this scoping review in order to identify sex- and gender-related factors that impact the lifecycle management of drugs processes. This review covered numerous topics, including clinical trial development, evidence on PK and PD processes, and ADRs. Here we report on sex- and gender-related factors in ADRs. 

### 3.1. Search Strategy

A health sciences librarian experienced in pharmacology conducted the search, which was sent to the research team for consultation. Using iterative feedback, a revised search was created, and the following databases were searched from inception to 23 July 2020: MEDLINE (Ovid), Embase (Ovid), Cochrane Library (Wiley), International Pharmaceutical Abstracts (Ovid), CINAHL (EBSCO), and Scopus. A search strategy using a combination of subject terms and keywords was used focusing on the concepts of Sex and Gender (including gender identity, sex factors, and sex characteristics) and the Drug Lifecycle (including research and development, discovery, design, legislation, costs, and industry). The search was translated as appropriate for each individual database and was restricted to studies on humans, written in the English language, and studies published in the last ten years. The full Ovid MEDLINE and Embase search is available in the Supplementary Table S1, and all searches are available upon request. Results from all databases were downloaded to Endnote X9, where they were duplicated before being uploaded to Rayyan for the screening process.

### 3.2. Literature Screening and Study Selection

Searches in six databases resulted in *n* = 8508 unique returns. Overall, five reviewers were involved in the screening process. First, titles and abstracts were screened independently by three reviewers for relevance. Then, the full texts of the articles were obtained and reviewed by two reviewers according to the inclusion criteria. English articles published between January 2010 and July 2020 with data from Canada, the USA, any country in the EU, and Australia were included. Studies that focused on the following were included: women, girls, men, boys, trans people/gender diverse people (all ages and other demographics within the defined populations); the lifecycle management of drugs; sex-related factors (e.g., biological, physiological, anatomical features, such as hormones, size, weight, metabolism, body parts, genetics, etc.), or gender-related factors such as roles, norms, relations, status, and identities affecting the lifecycle management of drugs. Studies on ADRs from national pharmacovigilance databases were included, while studies on ADRs with data collected from a provincial or local database were excluded. Additional exclusions were studies that focused on animals, pre-clinical trials, dietary supplements or any kind of natural health product, potentially inappropriate prescribing, adherence or non-adherence, sex/gender as a sociodemographic variable, cost of medications, blood products, or medical devices.

**Figure 1:**
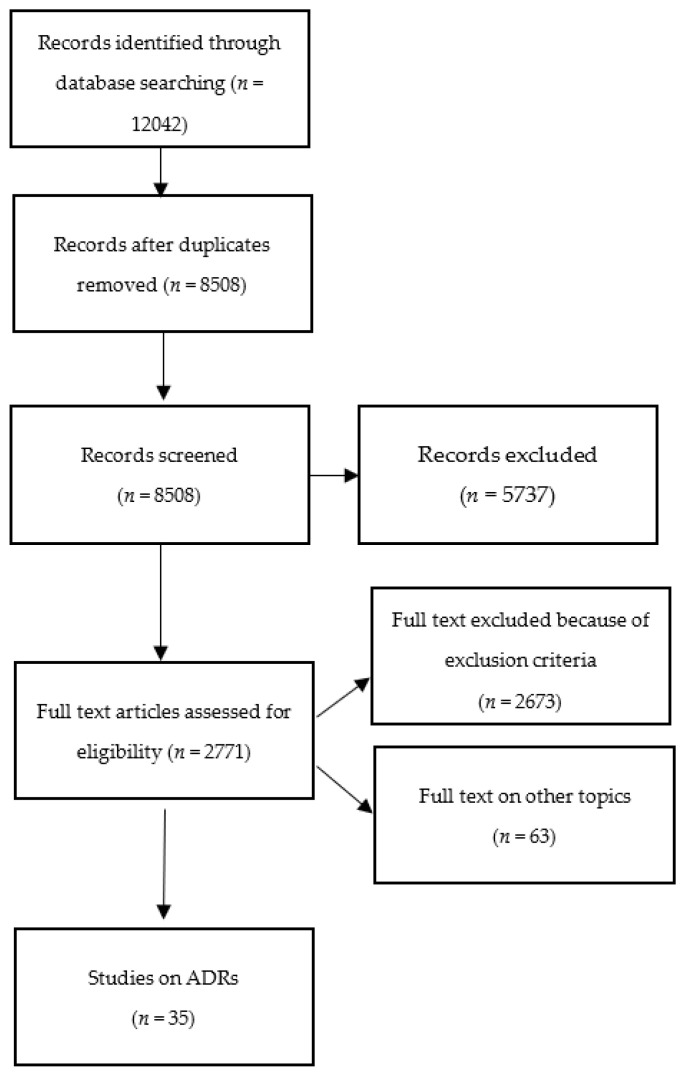
Screening process and included and excluded articles.

Before all screening phases, inclusion and exclusion criteria were calibrated among all reviewers. Inter-rater reliability (IRR) was monitored on a regular basis during title, abstract, and full-paper screening (after each quarter of the total retrieved papers) to ensure the reliability score (Cohen’s kappa) remained above κ = 0.6. The overall IRR was 0.63. After these three levels of screening, a total of *n* = 98 papers on lifecycle management of drugs processes were identified. Of those, *n* = 35 papers were on ADRs and therefore included in this scoping review. Figure 1 provides an overview of the literature search returns, the number of articles included and excluded at each level of screening, and the final number of included articles.

### 3.3. Data Extraction

Data from the included papers were extracted by one reviewer regarding the following information: author(s); year of publication; aim of the study; design; country; details on population; drugs investigated; medical condition(s); measured outcomes related to the lifecycle management of drugs; sex/gender analyses; main findings; limitations; and suggestions for future research.

### 3.4. Sex- and Gender-Based Analysis of the Included Papers

SGBA+ can be incorporated into research in several ways. Drawing upon an approach to SGBA+ in a systematic review of cannabis treatment outcomes [25], this scoping review focused on how the lifecycle management of drugs outcomes was analyzed and reported in relation to sex and gender. We created four categories: sex/gender-disaggregated outcomes (no testing for significance); sex/gender-disaggregated outcomes and testing for significance; sex/gender used as a confounder/controlled for (e.g., included in a regression model); and outcomes for one sex/gender group only. Table 1 presents this classification for each included study.

## 4. Results

### 4.1. Study Characteristics

Most of the included studies were conducted in the USA [33,37,38,39,40,41,43,45,46,48,56,57,58,59,60], followed by France [26,28,29,35,47,51], Sweden [36,54,55], the Netherlands [30,31,34], Portugal [42,52], the UK [44,49], Germany [32], Spain [50], Italy [27], and Australia [53]. Details of the country of origin of each study are presented in Table 1.

### 4.2. Drugs Associated with ADRs

A total of seven studies examined a variety of drugs, from a variety of drug classes associated with ADRs [27,31,32,36,42,44,60]. Sixteen studies addressed specific drug classes, such as antithrombotic drugs [26,55,56], vaccines [37,38,45], acne medication [57,58], alopecia medications [59], anticancer drugs [35], antihypertensives [54], antimalarial drugs [48], constipation or colonoscopy preparations [33], diabetes (Type 2) medications [30], and psychotropic drugs [34]. The remainder of the studies covered specific ADRs associated with a certain type of medication, such as neuromuscular blocking agents [47,51], phosphodiesterase type-5 inhibitors [40], nonsteroidal anti-inflammatory drugs (NSAIDs) [43], opioids [29], proton pump inhibitors [41], and antiepileptics [50]. Table 1 includes the drugs associated with ADRs.

### 4.3. ADR Reporting and Sex in Different Databases

Six articles examined sex differences from European databases [27,31,32,36,42,44], and one study was conducted with US data [60]. For example, data from a national Swedish pharmacovigilance database that were collected between 1 January 2008 and 31 December 2011 showed that regardless of seriousness, ADRs are reported more in women compared to men [36]. The authors used the Anatomical Therapeutic Chemical (ATC) classification and defined daily doses of prescribed medications to assess outcomes, which incorporated approximately 80% of prescribed therapies [36]. Authors reported crude reporting rates and rates standardized by the amount of dispensed drug and ATC level grouping, and further standardization was conducted after exclusion of sex-dependent therapies [36]. Individual case safety reports (ICSRs) were reported in 42% of men versus 57% of women. However, with standardization, there were more serious individual case safety reports (ICSRs) reported for men with a reporting rate of 0.30 in males versus 0.28 in females [36], with a footnote indicating a significant difference according to a 95% confidence interval. Similar results were found in a Portuguese study with data extracted from the central Portugal Regional Pharmacovigilance Unit from January 2001 to December 2009 [42]. While females have a higher proportion of ADRs overall, males were at a significantly increased risk of serious ADRs [42]. Evidence from the UK found that of 26,129 adverse event reports, significantly more females are represented in ADR reports than males [44]. Sex was missing from 3.6% of all reports [44]. 

An Italian study that examined sex differences in ADRs reported to the Italian National Network of Pharmacovigilance (INNP) between 2001 and 2016 for the most used Anatomic, Therapeutic, Chemical Classifications (ATCs) found that of the 301,233 ADR reports, 55.6% are from females, 43.1% males, and 1.3% did not indicate their sex [27]. The majority of ADRs are found in the 18–64 years age group and are mostly non-serious with a positive outcome. However, among serious reports, females have more, but death is more common in males than females. While males have more ADRs for two drug classes, females experience more ADRs for nine drug classes [27]. Interactions between age and sex were found among children, such that males younger than 2 years and older than 11 years have more ADRs, whereas females have a higher risk of ADRs between the age of 2 and 11 years [27]. In a German study, older age and sex together influenced patterns of reporting of ADRs [32]. There were more absolute reports of ADRs involving older females, increasing with age, but when considering 100,000 inhabitants or assumed drug-exposed inhabitants, more reports referred to older males [32]. 

In a US study using FDA Adverse Event Reporting System (FAERS) data from 2004 to 2011, Yu et al. (2016) found that of 668 drugs used in the 20 most frequent treatment regimens in the US, 307 drugs have sex differences in ADEs [60]. In a similar study with data from the National Pharmacovigilance Centre in the Netherlands, de Vries et al. (2019) explored sex differences in ADRs [31]. The final sample in the analysis consisted of 2483 distinct drug-ADR combinations, with 67% affecting females. Sex differences were found in 363 combinations (15%) and included 74 different drugs and 124 different ADRs. Females had a higher likelihood of reporting an ADR than males in most drug-ADR combinations (322 vs. 41), accounting for 89% of the cases [31]. 

In the Swedish Spontaneous Adverse Drug Event Reporting System (SWEDIS), the prevalence of bleeding reports from antithrombotic substances has been found to differ in females and males after adjusting for exposure to antithrombotics [55]. For example, even though clopidogrel was dispensed more to males, after adjustment for the number of patients exposed, reported bleeding events were higher in females [55]. However, the difference disappeared when adjusting for the number of prescriptions. The reported bleeding event risk from low-dose aspirin was lower in females, adjusted for patients exposed. For warfarin, no sex difference in bleeding event reports was found [55]. 

A US study found that the reporting quality of ADRs from oral antiplatelet agents (OAAs; aspirin, clopidogrel, prasugrel, ticagrelor, and vorapaxar) reports to the FAERS during 2015 was poor, with missing demographic data and under-reporting of co-use of aspirin with prasugrel or ticagrelor. Aspirin had less missing gender (although authors use gender, they meant sex) data than most other OAAs, except for prasugrel; however, age and gender (although authors use gender, they meant sex) data were predominantly missing when aspirin was the primary suspected drug of the ADR. US reports had less missing gender data (although authors use gender, they meant sex) than international reports, but more missing age data than international reports [56]. A French study found that the majority of gingival bleeding events occurred in females. The most frequently “suspected” drugs associated with gingival bleeding were antithrombotic (67.8%), especially fluindione, followed by other drugs such as furosemide, paracetamol, amiodarone, amoxicillin, paroxetine, ketoprofen, zolpidem, enalapril, and ramipril. The genitourinary system and sex hormones were involved in 11 cases (2.4%) [26]. A US study investigated the FAERS reports made in 2016 of gastrointestinal (GI) bleeding in older adults (65–100 years old) after taking nonsteroidal anti-inflammatory drugs (NSAIDs). Out of the 1347 cases, 51% affected males, and 49% affected females; however, neither age nor sex was found to be significant predictors of GI bleeding [43]. 

A US study investigated reports of alopecia associated with isotretinoin between 1997 and 2017 made to the FAERS. Of the 932 reports of alopecia, 98.4% of reports included gender information (although authors use gender, they meant sex), and of these, 68.7% were in females [58]. A different US study examined rates of pregnancy and pregnancy-related adverse events while taking isotretinoin between 1997 and 2017 reported to the FAERS. Of the pregnancy-related adverse event reports, 28.1% were abortions [57]. 

A US study investigated ADR reports to the FAERS database from two alopecia treatments, finasteride, and minoxidil, and found that for finasteride, which is only approved for use in males, 97.2% of ADE reports were in males, whereas 48.2% of minoxidil-related reports were from males [59]. Among males and females, finasteride was more frequently associated with adverse events in the reproductive system compared to minoxidil [59]. For males, erectile dysfunction was reported in 50.39% of cases of finasteride, whereas for minoxidil, it was reported in 4.35% of cases [59]. Females reported fetal toxicity and negative effects on the uterus. Males exposed to finasteride also reported psychiatric reactions, such as anxiety, depression, and cognitive disorder [59]. 

A French study investigated serious cutaneous ADRs associated with oral protein kinase inhibitors (PKIs), a class of oral cancer therapies, reported to the French Pharmacovigilance Database between 2008 and 2010. Of the 606 serious ADRs, most were related to skin and subcutaneous tissue disorders (*n* = 115; 19%), and of these, 63% were in male patients with cancer compared to 37% in females [35]. 

An investigation of reports to SWEDIS on ADRs associated with antihypertensive drugs reported between 1 July 2005 and 31 December 2012 found that sex differences in ADRs were reported for the 10 most commonly prescribed antihypertensive drugs. While women had a higher prevalence of ADRs from ACE inhibitors (ACE-I), angiotensin receptor blockers (ARB) combinations, thiazides, diuretics and potassium-sparing agents, and dihydropyridine calcium-channel-blockers (DHPs) with a potential linkage to dose exposure, men had a higher prevalence of ADRs from aldosterone antagonists, with no sex difference in dose exposure [54].

There is evidence from the FAERS that a syndrome class of neuropsychiatric ADRs from mefloquine, an antimalarial, is more common among males than females [48]. 

A US study that examined the renal ADRs from sodium phosphate tablets, a treatment for constipation and for colonoscopy preparation, found that 71% of the 178-sodium phosphate-containing colonoscopy tablet preparation reports were about or from females, with a mean body weight lower than the national average weight [33]. 

A study conducted in the Netherlands using data from the Dutch National Pharmacovigilance Center Lareb found that females reported ADRs significantly more often than males at 2 weeks (34% females vs. 25% males) and 6 weeks (37% vs. 28%) post metformin initiation, a drug used to treat type 2 diabetes [30]. Additionally, females were prescribed a significantly lower dose than males at the 9-month assessment [30]. The prescribed dose at initiation might explain why a higher proportion of females compared to males report an ADR in the case of metformin [61]. Indeed, the authors suggest that a smaller dose of metformin might benefit women at the beginning of the treatment [30].

There is also evidence of sex differences in the likelihood of reporting an ADR after receiving a vaccine, the seriousness of the report, the type of ADRs reported, and the reported symptoms. A UK study found that females reported a higher proportion of ADRs from vaccines than males [49]. This study also found patterns by sex and age in ADR reports from vaccines and other drugs, with reports from females most commonly occurring among those aged from 10 to 64 years and reports from males occurring in older age groups. The average age of those reporting ADRs from vaccines was similar in males (27 years) and females (28.9 years) [55]. In a US study among military personnel, females were found to report more ADEs in response to the monovalent pandemic 2009 (H1N1) vaccine than males [46]. However, in a different US study, the reporting rates of serious adverse events (SAEs) following yellow fever (YF) vaccination and specific SAEs such as YF vaccine-associated neurologic disease (YEL-AND), YF vaccine-associated viscerotropic disease (YEL-AVD), and anaphylaxis were higher among males [39]. In a US cross-sectional study on ADRs after receiving the trivalent influenza vaccine (FLU3), although 54% of the ADRs were reported by females and 43% were reported by males, males reported more symptoms than females. Sex was not included in 3% of reports [38]. 

Sex differences in reported symptoms were also found in a US study that investigated the Vaccine Adverse Event Reporting System (VAERS) database for ADRs following the human papillomavirus (HPV) vaccine reported between 2006 and 2017, where headaches and hypoesthesia occurred more often in females. This study also found interactions by age and sex whereby males were generally younger than females when they reported ADRs and experienced an increase in ADRs with older vaccination age [37]. In a different US study on adverse events and adenovirus vaccine among military personnel, analyses of the data collected between October 2011 and July 2018 suggest that among the 100 reports of ADRs, 72 were reported by males (the authors note that over 80% of U.S. military recruits are males and there were over 1.3 million doses administered) [45]. Thirty-nine reports were categorized as serious according to the MedDRA coding system, including 12 cases of Guillain Barré Syndrome (GBS). Of the 12 GBS reports, 2 were female, and 10 were male patients, with a median age of 22 years (17–28 range), and the median duration from vaccination to symptoms was 24 days (9–24 range). Prior to neurologic symptom onset, 8 of the 12 had a documented upper respiratory infection, and two had a documented diagnosis of Bell’s palsy [45].

Ekhart et al. (2018) [34] found that out of the 6791 ADR reports from selective serotonin reuptake inhibitors (SSRIs) reported by healthcare professionals and consumers between 1 January 2003 and 31 December 2016 to the Netherlands Pharmacovigilance Centre Lareb, 68% were in females. When all SSRIs were considered, between 62% and 82% of the reports concerned females. The authors explored if these outcomes could be explained by differences in the daily dose of SSRIs received; however, no differences between males and females were found. In the 48% of reports for which daily dose is available, males and females received the same daily doses of SSRIs with the exception of citalopram, where males received 25 mg and females received 22 mg [34].

In an Australian study on clozapine-induced myocarditis [53] conducted with data collected between January 1993 and December 2009, it was found that of the 10 fatal cases, 40% are female, and the mean age is 40 years (27–61 years) while among the non-fatal cases, 26% are female and the mean age is 38 years (21–73 years). Differences between fatal and non-fatal cases reveal no significant differences between sex, age, smoking status, mean dose of clozapine at onset, or concomitant use of sodium valproate. While smoking status did not significantly differ between fatal and non-fatal cases, seven out of nine fatal cases were in people who smoke, with data missing for one case. A significant difference in BMI was found, such that 60% of fatal cases have a BMI of greater than 30 kg/m^2^ (considered obese), whereas 26% of non-fatal cases had a BMI of greater than 30 kg/m^2^ (*p* = 0.025), and one case had a pre-existing cardiovascular disease [53]. 

In a Spanish cohort study on the association between Stevens–Johnson syndrome (SJS)/toxic epidermal necrolysis (TENS) and antiepileptics [50], there were 84 reports of SJS and 80 reports of TENS associated with 9 epileptic drugs. Of those reporting SJS, 59.5% are females and 38.1% males; for TENS, 55% are females and 43.8% males. For phenytoin, one of the most frequently involved antiepileptic drugs, ADRs are more common in older males, and with lamotrigine, ADRs are more common in younger females [50].

Findings from a French study on the drugs associated with memory disorders showed that memory disorders as an ADR were reported mostly in females (57%) [28]. Of the 30 drugs associated with memory impairment, zolpidem, topiramate, zopiclone, alprazolam, and bromazepam were the most common [28]. 

Two French studies found sex differences in anaphylaxis associated with neuromuscular blocking agents (NMBAs) [47]. Mertes et al. (2011) found that the incidence of anaphylaxis to NMBAs during anesthesia was higher for females compared to males [47]. Findings from Reitter et al. (2014) found that 36.4% of non-fatal anaphylaxis outcomes were in males, and 62.2% of the fatal anaphylaxis outcomes occurred in males. Male sex was found to be a significant risk factor for fatal outcome [51]. Amongst the 918 cases of anaphylaxis reported to the Portuguese Pharmacovigilance System, overall, there were more cases among females than males. Among adults, 70% of the ADRs involved women; however, there was a male predominance among the pediatric population (56%) [52]. 

Using the US FAERS, ten years of industry and non-industry reports of adverse cardiovascular events or mortality associated with three phosphodiesterase type-5 inhibitors (PDE5-i) (sildenafil, tadalafil, and vardenafil), used to treat erectile dysfunction, were investigated. Overall, 26,451 reports were reviewed, of which 14,818 ADRs were reported for sildenafil, 6085 for vardenafil, and 5548 for tadalafil. Cardiovascular ADRs were reported in 12% of reports and death in 8.2%. Sildenafil was associated with the most cardiovascular ADRs (76% versus 14% for tadalafil and 10% for vardenafil) as well as the most reported deaths (83.6% followed by tadalafil at 11%) [40].

Opioid-induced hypoglycemia appears to affect more women than men in the case of tramadol, codeine, and oxycodone, while it affects more men than women in the case of morphine, methadone, fentanyl, and buprenorphine [29]. 

In a US study on the association between the use of proton pump inhibitors (PPIs) (esomeprazole, lansoprazole, omeprazole, pantoprazole, rabeprazole, dexlansoprazole) and hypomagnesemia, Luk et al. (2013) found that of 66,102 subjects reporting one or more ADR while taking a PPI, 57.3% were females and 38.2% were males [41]. Sex was not available for 4.5% of records. For all six PPIs, there was a higher frequency of ADRs reported in females; however, the risk of female subjects experiencing hypomagnesemia was lower than for males [41]. 

## 5. Discussion

The objective of this review was to describe and synthesize sex- and gender-related ADR outcomes and analyze how sex and gender are considered in analyses and reporting on some major drug categories. This review is built upon research that reported on ADRs to several national pharmacovigilance systems by synthesizing sex- and gender-related ADR evidence considering the content, analyses, and reporting. The evidence from this scoping review on ADRs across different countries reveals that women report or are reported about in more ADRs compared to men. 

These observed sex differences in the number of reported ADRs can be linked to sex- or gender-related factors. Sex-related factors refer to biological differences between women and men, whereas gender-related factors refer to social, behavioral, or cultural differences [9]. Sex-related factors include a wide range of elements such as hormones, genetics, metabolic processes, anatomical characteristics, and organ function [62,63,64] and affect PK processes. Females, on average, weigh less than males; however, few drugs are administered based on weight, and if/when there is a “one size fits all” dose, the result will be a higher exposure among women [65]. 

This has clinical implications. For example, even though zolpidem was initially prescribed for both women and men at the same dose, given that women clear zolpidem more slowly than men, lower doses are currently recommended and prescribed for women [66]. Type A ADRs are predictable from the known pharmacology of a drug and are associated with high morbidity and low mortality [67], and they are dose dependent [31]. Type B reactions are idiosyncratic, bizarre, or novel responses that cannot be predicted from the known pharmacology of a drug and are associated with low morbidity and high mortality [67]. These distinctions may explain some of the findings in this scoping review. Although women reported more ADRs, men reported more serious ADRs. 

In addition to sex-related factors that affect both ADR occurrence and reporting, there are also gender-related factors such as gender roles, access to resources and opportunities, adherence to gender norms, degrees of commitment to dominant femininities and masculinities, and institutionalized inequities that reinforce sex and gender groups in all cultures and contexts. Women are more interested in and report much more active seeking of health-related information and receive more informal health-related information from close family members, other kin, and friends/workmates than men do [20]. However, in this scoping review, gender-related factors are not considered in drugs such as minoxidil [59], a drug used for alopecia treatment. This is despite the fact that hair loss may represent a significant aspect of women’s identity [68,69] and is perceived as related to femininity, which in turn affects the perception of attractiveness in society [68].

Most of the papers included in this review focus on a “sex differences” paradigm, even though pharmacokinetics and pharmacodynamics highlight processes of drug use and impact, thus requiring more fluid and complex conceptualizations of sex and gender. Sex and gender are unfortunately often used interchangeably without explicit definitions to ensure consistency of use. The quality of reporting is highly relevant, as missing data regarding sex, gender, and other variables, such as age, also affect the interpretation of the results and the data quality of the reporting. When intersections of sex and age are analyzed, the evidence shows different patterns for women and men across different ages. For example, both sex and age influence the differences found in ADRs from different drugs [27,32]. However, many studies only reported on age when conducting SGBA+ of the data, and there were no other intersectional variables in addition to sex. 

Drawing upon this scoping review, we recommend the following. First, an appropriate understanding of sex and gender concepts and the clinical relevance of sex- and gender-related factors in drug treatment and ADRs should be integrated into the standardized documents required for drug approval and pharmacovigilance databases. Regulatory agencies should include SGBA+ variables in the pharmacovigilance databases to enhance demographic reporting to reduce missing data and also consider relevant information such as PK/PD data analyzed during the drug approval process. They should build on well-known sex-related factors such as body weight, height, dose effect, age, etc., and other gender-related factors such as visits to healthcare providers, prevalence for prescriptions, etc. Including sex-related factors from PK/PD data into the pharmacovigilance databases will help to better understand the relationship between PK/PD processes and ADRs and reduce ADRs among females. By having relevant SGBA+ variables, data could be analyzed from an intersectional approach considering sex, age, and or race/ethnic background in assessing ADRs and PK/PD processes.

Second, sex-related factors related to PK and PD processes and the sex distribution involved in the testing of the drug should be added to the drug labels and monographs, so clinicians, consumers, and the public can be better informed. Additionally, regulatory agencies and pharmacovigilance databases should increase accessibility and ease of public reporting of adverse drug reactions and events, with increased system capacity for validation and accuracy and better systems to differentiate ADEs and ADRs.

## 6. Limitations

This review has limitations. We focused on ADRs from individual pharmacovigilance databases across several countries, and the lack of standardization between systems might limit the comparability of the data. There are different regulations concerning reporting, with many voluntary-based systems, and different terminologies. Even though the International Council for Harmonisation of Technical Requirements for Registration of Pharmaceuticals for Human Use defines an AE as “any untoward medical occurrence that may present during treatment with a pharmaceutical product but which does not necessarily have a causal relationship with this treatment” [70] and an ADR as “a response to a drug which is noxious and unintended and which occurs at doses normally used in man for prophylaxis, diagnosis, or therapy of disease or for the modification of physiologic function” [70], ADRs and AEs were used interchangeably. Furthermore, most of the studies were conducted in higher-income countries such as the United States, the United Kingdom, Australia, and the European Union. These results may not be wholly applicable to other countries due to a lack of knowledge on ADR reporting structures, availability of resources for reporting, and management strategies for ADRs. Further, it is often unknown what the denominator of usage of various drugs is, or the number of prescriptions by sex or subgroup, against which ADRs are reported.

## 7. Conclusions

This scoping review shows that there are differences in the reporting of ADRs by and about women and men across countries such as the USA, Canada, the UK, Australia, and countries from the EU. Tying specific adverse drug reports or clusters to specific drugs or combinations of drugs is challenging, given the types and forms of data available. Similarly, tying adverse events to different stages in therapeutic use poses even more challenges to fully understanding sex-related factors affecting ADRs and gender-related factors linked to reporting practices. Accruing enough information via adverse event reporting to determine sex-linked mechanisms, dosage and underlying conditions, accuracy of diagnosis, interactions with recreational or over-the-counter drugs, or other clinical or environmental factors remains elusive given current data collection systems. All these factors are affected by sex and gender, making an SGBA+ of adverse event reporting and reports, while essential, a challenge. While translating these events into improvements in clinical practice and enhanced public information is therefore problematic, it is also necessary in order to create changes in pharmacovigilance systems and improve our collective understanding of ADR distributions among sexes. 

By and large, the adverse drug reports do not apply PK/PD principles to their descriptions of ADRs, and these studies draw conclusions that do not engage with sex (and age)-related factors affecting PK/PD processes. Further, the authors may not know or report the numbers of prescriptions for a given drug when analyzing a database of adverse events, making some kinds of analyses impossible and denominators unknown. They may also not know the rates of diagnoses in a given population group, against which to compare rates of prescribing or adverse events. Given that national databases rely on voluntary reporting, caution about interpretation and use of such data due to self-reports, subjectivity, under-reporting, invalidated claims, lack of verification, incompleteness, and little analysis of causation, there is considerable room for improvement in systematically addressing adverse event reporting that would assist in understanding the impact of sex, gender, age, and other interrelated factors that coordinates evidence on sex-related pharmacokinetics and pharmacodynamics.

In short, many national pharmacovigilance systems for ADRs are partial, difficult to compare directly, and inconsistently include sex-related data. They do not typically link their reports to pharmacokinetic or pharmacodynamic principles and processes. They rarely include gender-related data. A consistent sex- and gender-based analysis plus (SGBA+) of national systems would improve designs and serve to produce ADR data that would improve the health of all, increase comparability, and in particular, redress historical oversights in protecting women’s health.

## Figures and Tables

**Table 1:** Characteristics of the included studies.

Author(s) and Year	Country	Study Design	Research Aim	Drug(s)	SGBA+
Bondon-Guitton et al. (2017) [26]	France	Cross-sectional	To identify the drugs most frequently suspected in the occurrence of gingival bleeding	Discovering which drugs are associated with gingival bleeding was the main outcome. Among drugs known to increase the risk of gingival bleeding, the most frequently involved were fluindione, furosemide, paracetamol, amiodarone, amoxicillin, paroxetine, or ketoprofen. The authors also identified signals for drugs not usually known to be involved in bleeding, like zolpidem, enalapril, or ramipril	Sex-disaggregated outcomes (no testing for significance)
Castellana et al. (2018) [27]	Italy	Cross-sectional	To investigate the gender-related differences in adverse drug reactions (ADRs) in the Italian population, on the basis of sex, during a 15-year observation period	The most-reported ATCs (Anatomic, Therapeutic, Chemical Classification): protease inhibitors, reverse transcriptase nucleoside inhibitors, thyroid hormones, aminoquinoline antimalarials, COX-inhibitor anti-inflammatory and antirheumatic drugs, selective serotonin reuptake inhibitor antidepressants, benzodiazepine derivative anxiolytics, acetic acid derivative anti-inflammatory and antirheumatic drugs and related substances, broad-spectrum penicillins, penicillin associations including beta-lactamase inhibitors and propionic acid derivative anti-inflammatory and antirheumatic drugs	Sex-disaggregated outcomes (%) (no testing for significance)
Chavant et al. (2011) [28]	France	Cross-sectional	To examine which drugs are associated with memory disorders	The main therapeutic classes suspected were hypnotics (76 cases), anticonvulsants (68 cases), anxiolytics (66 cases), antidepressants (55 cases), analgesics (45 cases), and antipsychotic drugs (29 cases)	Sex-disaggregated outcomes (no testing for significance)
Chretien et al. (2019) [29]	France	Cross-sectional	To determine if drug-induced hypoglycemia could be a class effect for opioids	Opioids (codeine, fentanyl, hydromorphone, methadone, morphine, oxycodone, tramadol, buprenorphine, and nalbuphine)	Sex-disaggregated outcomes (no testing for significance)
De Vries et al. (2020) [30]	Netherlands	Cohort study (prospective or retrospective)	To assess whether sex differences in reported adverse drug reactions (ADRs) for metformin are observed at different times after initiation and to explore their concurrence with sex differences in the dose of metformin over time	Metformin	Sex-disaggregated outcomes and testing for significance
de Vries et al. (2019) [31]	Netherlands	Cross-sectional	To assess sex differences in adverse drug reactions (ADRs) reported to the national pharmacovigilance center in the Netherlands, taking sex differences in the number of drug users into account. The secondary aims of this study were to assess for which drugs and for which ADRs sex differences were identified most often	74 different drugs from many different classes were identified as having a potentially significant sex difference in experiences ADRs	Sex-disaggregated outcomes and testing for significance
Dubrall et al. (2020) [32]	Germany	Cross-sectional	The first aim of the study was to determine the number of ADR reports regarding older adults (>65 years) and to set these reports in relation to (i) the number of spontaneous ADR reports regarding younger adults (19–65) and (ii) the number of inhabitants and assumed drug-exposed inhabitants, and to oppose the ADR reports to the number of defined daily doses (DDD) used per insured person. The second aim was to analyze if some of the reported characteristics are more often described in the ADR reports of older adults compared to younger adults	The ten drug classes most frequently suspected in older adults: antithrombotic agents, antineoplastic agents, antiphlogistics and antirheumatics, systemic antibiotics, agents acting on the renin–angiotensin system, psycholanaleptics, psycholeptics, lipid-modifying agents, antidiabetics, and analgesics. The ten drug classes most frequently suspected in psycholeptics, systemic antibiotics, antineoplastic agents, psychoanaleptics, immunostimulants, antithrombotic agents, immunosuppressives, sex hormones, antiepileptics, antiphlogistics, and antirheumatics	Sex-disaggregated outcomes and testing for significance
Ehrenpreis et al. (2011) [33]	USA	Cross-sectional	To analyze renal risks of sodium phosphate tablets, especially the role of body weight and gender as risk factors for renal complications	Sodium-phosphate-containing colonoscopy preparations, such as sodium phosphate tablets sold as OsmoPrep and Visicol, as well as polyethylene glycol (PEG) colonoscopy preparations	Sex-disaggregated outcomes (no testing for significance)
Ekhart et al. (2018) [34]	Netherlands	Cross-sectional	To investigate whether reports of adverse drug reactions (ADRs) when using selective serotonin reuptake inhibitors (SSRIs) concern women and men equally in the database of the Netherlands Pharmacovigilance Centre Lareb, taking into account the differences in the number of users. The secondary aim was to explore if differences could be explained by the daily dosage received of the SSRIs under study	Selective serotonin reuptake inhibitors (SSRIs): citalopram, escitalopram, fluoxetine, fluvoxamine, paroxetine, sertraline, and venlafaxine. Venlafaxine in daily doses up to 150 mg can be regarded as an SSRI	Sex-disaggregated outcomes and testing for significance
Faye et al. (2013) [35]	France	Cohort study (prospective or retrospective)	To describe all ADRs for oral protein kinase inhibitors, their characteristics, and whether they were labeled	Protein kinase inhibitors (erlotinib, gefitinib, imatinib, nilotinib, dasatinib, sunitinib, sorafenib, pazopanib, and lapatinib)	Sex-disaggregated outcomes and testing for significance
Holm et al. (2017) [36]	Sweden	Cross-sectional	To investigate how reporting of adverse drug reactions (ADRs) among adults in Sweden is associated with age and sex, in addition to seriousness of the reaction and drug utilization	Several ATC: blood and blood forming organs, cardiovascular system, general anti-infectives for systemic use, nervous system and a fifth composite group, consisting of suspected drugs belonging to the remaining ATC codes	Sex-disaggregated outcomes and testing for significance
Jia et al. (2019) [37]	USA	Cohort	To evaluate the safety profiles of human papillomavirus (HPV) vaccines with regard to the distribution of adverse events (AE) across gender and age, and the correlations across various AEs using the Food and Drug Administration/Centers for Disease Control and Prevention Vaccine Adverse Event Reporting System (VAERS). Research questions: (1) Are the frequencies of AEs different across different gender and age groups? If significant differences are observed, it is essential to develop a more precise vaccine information statement for these targeted subgroups. (2) Are there any correlations among the AEs? Specifically, we explored whether some AEs were more likely to occur together	Human papillomavirus (HPV) vaccine (vaccine types: HPVX/HPV2/HPV4/HPV9 (HPV vaccine with no brand name/HPV Cervarix/HPV Gardasil/HPV Gardasil 9))	Sex-disaggregated outcomes and testing for significance
Jingcheng et al. (2017) [38]	USA	Cross-sectional	To study individual differences, considering sex and age, in adverse reactions following vaccination of the trivalent influenza vaccine	Trivalent influenza virus vaccine (FLU3)	Sex-disaggregated outcomes and testing for significance
Lindsey et al. (2016) [39]	USA	Cross-sectional	To describe adverse events following yellow fever (YF) vaccination reported to the U.S. Vaccine Adverse Event Reporting System (VAERS) from 2007 to 2013 and to calculate age- and sex-specific reporting rates of all serious adverse events (SAE), anaphylaxis, YF-vaccine-associated neurologic disease (YEL-AND) and YF-vaccine-associated viscerotropic disease (YEL-AVD)	Yellow fever vaccine	Sex-disaggregated outcomes (no testing for significance)
Lowe & Costabile (2012) [40]	USA	Cross-sectional	To document the rate of reported significant adverse cardiovascular events or mortality associated with each of three phosphodiesterase type-5 inhibitors (PDE5-I) used to treat erectile dysfunction over 10 years by a review of industry and non-industry reports submitted to the FDA	Sildenafil, tadalafil, and vardenafil	Outcomes for one sex/gender group only—males
Luk et al. (2013) [41]	USA	Cohort	To examine the association between use of different proton pump inhibitors (PPIs) and hypomagnesemia by examining frequency of occurrence among reported ADRs from the FDA AERS database	Proton pump inhibitors (esomeprazole, lansoprazole, omeprazole, pantoprazole, rabeprazole, and dexlansoprazole)	Sex/gender used as a confounder/controlled for (e.g., included in a regression model)
Macedo et al. (2011) [42]	Portugal	Cross-sectional	To evaluate the role of multiple drug exposure as an independent risk factor for serious ADRs and to validate the hypothesis of a trend for increased seriousness of ADRs in the presence of an increased number of simultaneous drug exposures	The drugs most commonly reported as responsible for the suspected ADRs were anti-infectives for systemic use (including vaccines; *n* = 452; 30.5%), drugs active on the muscle-skeletal system (*n* = 257; 17.3%), the nervous system (*n* = 240; 16.2%) and the cardiovascular system (*n* = 210; 14.2%). Together they accounted for 78.2% of all ADRs	Sex-disaggregated outcomes and testing for significance Sex/gender used as a confounder/controlled for (e.g., included in a regression model)
McDonald et al. (2019) [43]	USA	Cross-sectional	To identify predictors of gastrointestinal (GI) bleeding in older adults (65–100 years) when a nonsteroidal anti-inflammatory drug (NSAID) was identified as the primary suspect for an adverse drug event (ADE)	Nonsteroidal anti-inflammatory drug (NSAID)	Sex-disaggregated outcomes and testing for significance
McLernon et al. (2010) [44]	UK	Cohort study	To compare patient characteristics, suspected drugs, and suspected adverse reactions (ADRs) reported by patients with those reported by healthcare professionals using the Yellow Card Scheme (YCS)	The 20 most frequent suspect drugs reported: simvastatin, paroxetine, atorvastatin, diclofenac, amlodipine, venlafaxine, citalopram, tramadol, cyproterone and estrogen, trimethoprim, erythromycin, fluoxetine, ibuprofen, atenolol, olanzapine, omeprazole, bendroflumethiazide, paracetamol, combinations excluding psycholeptics, risperidone	Sex-disaggregated outcomes and testing for significance
McNeil et al. (2019) [45]	USA	Cross-sectional	To evaluate the safety profile of the adenovirus vaccine by reviewing reports submitted to the Vaccine Adverse Event Reporting System (VAERS)	Adenovirus vaccination	Sex-disaggregated outcomes and testing for significance
McNeil et al. (2012) [46]	USA	Cross-sectional	The aim of this pharmacovigilance study was to examine the spectrum of adverse events among reservists in a US military unit after receiving monovalent pandemic 2009 (H1N1) vaccine (MIV) and to investigate the factors contributing to a cluster of reports to the Vaccine Adverse Event Reporting System (VAERS) that occurred on 20 February 2010 from members of this unit	Monovalent pandemic 2009 (H1N1) vaccine (MIV)	Sex-disaggregated outcomes and testing for significance
Mertes et al. (2011) [47]	France	Cohort study	To report the results of an 8-year survey of anaphylaxis during anesthesia in France	The ADRs were associated to NMBAs (*n* = 539; 31.97%) and antibiotics (*n* = 511; 9.02%)	Sex-disaggregated outcomes and testing for significance
Nevin & Leoutsakos (2017) [48]	USA	Case control	To identify a distinct neuropsychiatric syndrome class associated with reports of adverse reactions from mefloquine use, to confirm the association of this syndrome with prodromal symptoms, and to identify other specific symptoms commonly associated with it that might inform improvements in case findings	Mefloquinem atovaquine-proguanil, doxycycline, chloroqine, and loperamide	Sex-disaggregated outcomes
O’Donovan et al. (2019) [49]	UK	Cross-sectional	To analyze a large sample of patient Yellow Card reports from July to December 2015. Objectives were to (1) describe all patient reports submitted to the Medicines and Healthcare Regulatory Agency (MHRA) over a 6-month period in terms of reporter characteristics, drugs, reactions, and outcomes; (2) explore factors associated with reports classed by the MHRA as serious; and (3) compare selected parameters to the analysis of reports from the first 2 years	Vaccines and other drugs	Sex- and age-disaggregated outcomes
Ordonez et al. (2015) [50]	Spain	Cohort	To assess the association between Stevens–Johnson Syndrome (SJS)/toxic epidermal necrolysis (TENS) and antiepileptics, including the most recently authorized drugs, based on the information provided by the spontaneous reporting of suspected adverse drug reactions (ADR)	Antiepileptic drugs (phenytoin, lamotrigine, carbamazepine, valproate, phenobarbital oxcarbazepine, levetiracetam, primidone, and gabapentin)	Sex-disaggregated outcomes and testing for significance
Reitter et al. (2014) [51]	France	Cohort study (prospective or retrospective)	To evaluate the mortality rate in France from anaphylactic reactions to neuromuscular blocking agents (NMBAs), to identify risk factors for a fatal outcome, and to describe management of the cases that proved fatal	Neuromuscular blocking agents (NMBAs): atracurium, cisatracurium, mivacurium, pancuronium, rocuronium, sux-amethonium, and vecuronium	Sex/gender used as a confounder/controlled for (e.g., included in a regression model)
Ribeiro-Vaz et al. (2013) [52]	Portugal	Case series	To characterize a case series of anaphylactic reactions reported to the Portuguese Pharmacovigilance authority during the past decade	Drugs associated with the reporting cases: antibiotics (17%), nonsteroidal anti-inflammatory drugs/acetaminophen (13%), antineoplastic/cytotoxic drugs, immune-modulators, vaccines, and radiographic contrast media	Sex-disaggregated outcomes and testing for significance
Ronaldson et al. (2011) [53]	Australia	Cohort	To compare key characteristics between clozapine-induced myocarditis fatal and non-fatal cases and to identify factors that may serve as clues to the prevention of myocarditis-related fatality in patients starting clozapine	Clozapine	Sex-disaggregated outcomes and testing for significance
Rydberg et al. (2018) [54]	Sweden	Cross-sectional	To explore sex differences regarding reported adverse drug events (ADEs) from the 10 most commonly prescribed antihypertensive medicines in Sweden, using the Swedish Spontaneous Adverse Drug Event Reporting System (SWEDIS) and the Swedish Prescribed Drug Register (SPDR)	10 selected groups of antihypertensives; ACE-Is, ACE-I/thiazide combinations, ARBs, ARB/thiazide combinations, Thiazides, diuretics and potassium-sparing agents, sulfonamides, aldosterone antagonists, dihydropyridines, and beta blockers	Sex-disaggregated outcomes and testing for significance
Rydberg et al. (2014) [55]	Sweden	Cross-sectional	To analyze sex differences in reported bleeding events of warfarin, low-dose aspirin, and clopidogrel in Swedish Spontaneous Adverse Drug Event Reporting System (SWEDIS), adjusted by drug utilization data from the Swedish Prescribed Drug register	Warfarin, low-dose aspirin, and clopidogrel	Sex-disaggregated outcomes and testing for significance
Serebruany et al. (2017) [56]	USA	Cross-sectional	To assess the quality and completeness of aspirin and other oral antiplatelet agents (OAAs) cases reported to the U.S. Food and Drug Administration (FDA) Adverse Event (AE) Reporting System (FAERS) in terms of age and gender	Oral antiplatelet agents (OAAs): Aspirin, clopidogrel, prasugrel, ticagrelor, and vorapaxar	% gender and age missing from reports of AEs.
Tkachenko et al. (2019) [57]	USA	Cross-sectional	To determine the frequency and rate of pregnancy and pregnancy-related adverse events among women taking isotretinoin reported to the US FDA. The authors were interested in understanding how AEs might change in the age of iPLEDGE, a program initiated in 2006 to reduce fetal exposure to isotretinoin as it carries a risk of teratogenesis	Isotretinoin	Outcomes for one sex/gender group only—females
Tkachenko et al. (2019) [58]	USA	Cross-sectional	To investigate the frequency of FDA reports of alopecia for patients taking isotretinoin from 1997 to 2017, with attention to age and gender differences	Isotretinoin	Sex-disaggregated outcomes (no testing for significance)
Wu et al. (2016) [59]	USA	Cross-sectional	To examine the clinical reports submitted to FAERS from 2004 to 2014 to compare the adverse effects of finasteride and minoxidil, the only 2 FDA-approved alopecia drugs	Finasteride (approved only in males for alopecia) and minoxidil	Sex-disaggregated outcomes and testing for significance
Yu et al. (2016) [60]	USA	Cross-sectional	To assess the extent of sex differences in ADEs across a wide range of treatments, to identify the drugs that show significant sex differences in 20 treatment regimens and 668 specific drugs, and to pinpoint the specific ADEs that account for the observed sex differences in the effects of these drugs	Antihypertensives, lipid-regulating agents, antidepressants, antiulcer agents, narcotic analgesics, antidiabetics, thyroid agents, antiepileptics, contraceptives, respiratory system agents, anticoagulants ADHD agents, insomnia agents, benign prostate hyperplasia agents, antipsychotics, osteoporosis agents, overactive bladder agents, antiparkinsonian agents, antimigraine agents, and Alzheimer agents	Sex-disaggregated outcomes and testing for significance

## Data Availability

Data sharing not applicable.

## Supplementary Materials

The following supporting information can be downloaded at: https://www.mdpi.com/article/10.3390/ph15030298/s1, Table S1: Database(s): Embase from 1974 to 22 July 2020, Ovid MEDLINE(R) ALL 1946 to 22 July 2020.
