# Supplementary material for: A Sex- and Gender-Based Analysis of Adverse Drug Reactions: A Scoping Review of Pharmacovigilance Databases
Source: Pharmaceuticals (Basel). 2022 Feb 28;15(3):298. doi: 10.3390/ph15030298 (PMC8950058; doi:10.3390/ph15030298)
Supplement: Supplementary file 1 [file pharmaceuticals-15-00298-s001.zip › pharmaceuticals-1604499-supplementary.pdf]

Table S1. Database(s): Embase 1974 to 2020 July 22, Ovid MEDLINE(R) ALL 1946 to July 22, 2020.

#	Searches	Results
1	exp Gender Identity/	36797
2	Sex Factors/	270976
3	Sex Characteristics/	55645
4	(sex adj5 biological variable$).tw,kf.	404
5	(sex adj2 gender adj3 based).tw,kf.	276
6	(SABV$ or SGBA$).tw,kf.	91
7	(gender adj2 (main stream$ or mainstream$)).tw,kf.	223
8	((sex$ or gender$) adj3 (analy$ or aspect$ or based or bias$ or characteristic$ or comparison$ or determinant$ or differen$ or dimorphism$ or di-morphism$ or disparit$ or distribut* or equalit$ or equity or factor$ or identit$ or inequit$ or inequalit$ or informed$ or institutional$ or integrated or interaction* or issue$ or medicine or minorit* or norm or norms or related or responsiv$ or role$ or specific$ or transform$)).tw,kf.	639634
9	or/1-8 [Sex and Gender Concept]	893598
10	exp Drug Industry/	127186
11	drug development/ or exp drug approval/ or exp drug design/ or drug evaluation/ or drug evaluation, preclinical/ or drug repositioning/	489293
12	Drug Discovery/	51759
13	Drug Costs/	90180
14	legislation, drug/ or "drug and narcotic control"/ or drug approval/ or drug recalls/ or safety-based drug withdrawals/	93734
15	product surveillance, postmarketing/ or adverse drug reaction reporting systems/ or clinical trials, phase iv as topic/ or pharmacovigilance/ or prescription drug monitoring programs/	29677
16	Precision Medicine/	36854
17	exp "Drug-Related Side Effects and Adverse Reactions"/	645062
18	((drug$ or pharm$) adj3 (lifecycle$ or life cycle$)).tw,kf.	658
19	(precision adj (medicine or pharm$)).tw,kf.	22449
20	pharmacovigilan$.tw,kf.	15321
21	((drug$ or pharm$) adj2 (approv$ or cost$ or develop$ or design$ or discover$ or disposal$ or evaluat$ or industr$ or legislat$ or manufacture$ or market$ or registrat$ or regulat$ or recall$ or reposition$ or re-position$ or research$ or policy or policies or service$)).tw,kf.	508036
22	or/10-21 [Drug Life Cycle Concept]	1765219
23	9 and 22	11336
24	limit 23 to yr="2010 -Current"	7195
25	exp Animals/ not (exp Animals/ and Humans/)	16703431
26	24 not 25	4273
27	limit 26 to english language	4158
28	27 use medall	1835
29	exp gender identity/	36797

30	exp sex difference/	429582
31	"gender and sex"/	1034
32	(sex adj5 biological variable$).tw,kw.	404
33	(sex adj2 gender adj3 based).tw,kw.	274
34	(SABV$ or SGBA$).tw,kw.	98
35	(gender adj2 (main stream$ or mainstream$)).tw,kw.	231
36	((sex$ or gender$) adj3 (analy$ or aspect$ or based or bias$ or characteristic$ or comparison$ or determinant$ or differen$ or dimorphism$ or di-morphism$ or disparit$ or distribut* or equalit$ or equity or factor$ or identit$ or inequit$ or inequalit$ or informed$ or institutional$ or integrated or interaction* or issue$ or medicine or minorit* or norm or norms or related or responsiv$ or role$ or specific$ or transform$)).tw,kw.	641489
37	or/29-36 [Sex and Gender Concept]	942005
38	drug industry/ or drug packaging/ or drug labeling/	142880
39	drug development/ or drug design/ or polypharmacology/ or drug approval/	204562
40	drug analysis/ or drug determination/ or drug discrimination/ or drug repositioning/	194578
41	"drug cost"/ or drug legislation/	116176
42	drug control/ or drug approval/ or drug formulary/ or drug legislation/ or drug program/ or drug recall/	98797
43	exp postmarketing surveillance/ or exp pharmacovigilance/ or "phase 4 clinical trial (topic)"/ or prescription drug monitoring program/	41434
44	Precision Medicine/	36854
45	adverse drug reaction/	270252
46	((drug$ or pharm$) adj3 (lifecycle$ or life cycle$)).tw,kw.	679
47	(precision adj (medicine or pharm$)).tw,kw.	22594
48	pharmacovigilan$.tw,kw.	16509
49	((drug$ or pharm$) adj2 (approv$ or cost$ or develop$ or design$ or discover$ or disposal$ or evaluat$ or industr$ or legislat$ or manufacture$ or market$ or registrat$ or regulat$ or recall$ or reposition$ or re-position$ or research$ or policy or policies or service$)).tw,kw.	516268
50	or/38-49 [Drug Life Cycle Concept]	1360829
51	37 and 50	10638
52	limit 51 to yr="2010 -Current"	6628
53	(rat or rats or mouse or mice or swine or porcine or murine or sheep or lambs or pigs or piglets or rabbit or rabbits or cat or cats or dog or dogs or cattle or bovine or monkey or monkeys or trout or marmoset$1).ti. and animal experiment/	1068382
54	Animal experiment/ not (human experiment/ or human/)	2256117
55	53 or 54	2304562
56	52 not 55	6409
57	56 use oemezd	5261
58	28 or 57	7096
59	limit 58 to yr="1860 - 2015"	3196

60 limit 58 to yr="2016 - Current"	3900
61 remove duplicates from 59	2802
62 remove duplicates from 60	3370
63 61 use medall	891
64 62 use medall	937
65 61 use oemezd	1911
66 62 use oemezd	2433
67 63 or 64	1828
68 65 or 66	4344
